# 微波消解-ICP-MS法测定肺癌患者体液和头发中的14种元素的含量

**DOI:** 10.3779/j.issn.1009-3419.2010.08.13

**Published:** 2010-08-20

**Authors:** 琳琳 窦, 文龙 许

**Affiliations:** 311201 杭州，浙江萧山医院检验科 Department of Clinical Laboratory, Zhejiang Xiaoshan Hospital, Hangzhou 311201, China

**Keywords:** 微波消解, ICP-MS, 肺肿瘤, 体液, 毛发, 元素含量测定, Microwave digestion, ICP-MS, Lung neoplasms, Body fluid, Hair, Content determination of inorganic elements

## Abstract

**背景与目的:**

体液和组织中的微量元素的含量是多种疾病的重要监测指标，对肺癌病人体液和头发中的微量元素进行分析，有助于肺癌的早期诊断及治疗效果评价。本研究旨在发展一种能够测定肺癌患者体液和毛发中Cr、Fe、Mn、Al、Cd、Cu、Zn、Ni、Se、Pb、Ca、Mg、Sr、P等14种微量元素的含量的方法。

**方法:**

采用微波消解法对取自16例肺癌患者的48份样品进行消解，利用ICP-MS方法进行元素含量测定。

**结果:**

利用GBW09101人发成分分析标准物质对所建方法的准确性进行了考察，结果表明，测得值与标准值之间不存在统计学差异。利用所建的方法分析了来自16例肺癌患者的48份样品，从中发现了若干规律。

**结论:**

本方法适用于肺癌患者体液和毛发中微量元素的测定，对于肺癌的诊断和治疗具有一定的临床意义。

## Abstract (en)

**Background and objective:**

The trace element contents in the body fluid and hair are the important monitoring indicators for many diseases. The analysis of the trace element contents in the samples of lung cancer patients is helpful to the early diagnosis and treatment effectiveness evaluation to the patients. The aim of this study is to develop an ICPMS method for the determination of Cr, Fe, Mn, Al, Cd, Cu, Zn, Ni, Se, Pb, Ca, Mg, Sr, P in the body fluid and hair of lung cancer patients.

**Methods:**

Samples of body fluid and hair from lung cancer patients were digested with microwave and 14 trace elements were determined with inductively coupled plasma mass spectrometry.

**Results:**

GBW09101 standard reference material of human hair was used to validate the accuracy of the established method, and the results indicated that there is no obvious difference between the measured values and the references values. Forty-eight samples from 16 lung cancer patients were analyzed with the established method, and several generalizations were discovered.

**Conclusion:**

The established method can be used for the multielement simultaneous determination of the samples of lung cancer patients, which are helpful to the diagnosis and treatment of the lung cancer.

癌症与微量元素之间存在着十分复杂而又密切的关系，某些微量元素的过量或缺乏，有可能成为肿瘤发生的病因。同时，癌症的发生和发展，会引起机体内的微量元素发生变化。这种变化将有可能被人们用来诊断或预报癌症^[1-3]^。多篇文献^[4-6]^报道了肺癌患者体液、毛发及组织中微量元素含量的测定方法，但所采用的方法多为原子光谱方法，且样品前处理较为繁琐。本文发展了一种微波消解-ICP-MS的分析方法，可同时用于肺癌患者的毛发、血液和尿液中Cr、Fe、Mn、Al、Cd、Cu、Zn、Ni、Se、Pb、Ca、Mg、Sr、P等14种微量元素的测定。并对所建分析方法的方法学参数进行了考察。

## 1 材料与方法

### 1.1 试剂与样品

HNO_3_（分析纯HNO_3_为原料，亚沸蒸馏6次制得）、H_2_O_2_、Agilent多元素混标Part # 518324688及内标Part # 18324680溶液。人发成分分析标准物质GBW09101（购自天津华科瑞丰标准物质技术服务有限公司）。

毛发、血液、尿液样品来源于本院已确诊为肺癌的患者，由医院专人负责收集，共采集16例肺癌患者的血液、尿液和毛发样品48份。为了进行比较，另外采集来自正常人的样品15份，共计63份样品，放入-80 ℃冰箱冷冻保存。

### 1.2 仪器及操作参数

7500a型电感耦合等离子体质谱仪（ICP-MS）（美国Agilent公司）；Speed wave MW-3微波消解系统（德国Berghof公司）；BSB-939-IR酸蒸馏纯化器（德国Berghof公司）；Milli-Q超纯水系统（美国Millipore公司）；AJ100型电子天平（瑞士Mettler-Toledo公司）。

微波消解样品采用Speed wave MW-3^+^微波消解系统中自带的LEAF消解程序，按照100 ℃–180 ℃–200 ℃–210 ℃的步骤进行升温，每次升温过程时间为2 min，升温后在每个温度下均保持10 min，因此，整个消解过程历时48 min。经优化后，所选用的ICP-MS仪器工作参数列于表 1中。

**1:** Agilent 7500a ICP-MS仪器工作参数 The operation parameters of Agilent 7500a ICP-MS

Parameters	Running conditions	Parameters	Running conditions
Radio-frequency power	1 500 W	sample uptake flow rate	1.222 *μ*L/min
Sampling depth	6.0 mm	analytical model	quantitative analysis
Carrier gas flux	1.0 L/min	residence time	30 s
Aux gas flux	1.0 L/min	scan mode	Peak Hopping
Plasma gas flux	16.0 L/min	points/ mass	5
Skimmer cone antilinear	0.8 mm	integral time	0.1 s
Sampling cone antilinear	1.0 mm	data collecting repeat	5

### 1.3 样品消解方法

准确称量0.5 g样品，放入消解罐中，加入5 mL高纯HNO_3_和0.3 mL 5 mg/kg的In元素内标溶液，虚掩罐盖进行预消解约5 h，以排出HNO_3_与样品反应所生成的气体，然后将消解罐旋紧，放入微波炉中，按照LEAF消解程序设定的步骤进行升温消解，消解结束，待消解罐冷却后，拧开，加入1 mL H_2_O_2_，待样品变为无色透明溶液后，用超纯水定容至30 mL，进行分析。每批次的消解均包括3份GBW09101人发成分分析标准物质样品，用以验证分析结果的准确性。

### 1.4 标准溶液

以体积分数5%的高纯HNO_3_作为空白样品，分别利用Agilent公司多元素混标配制Cr、Fe、Mn、Al、Cd、Cu、Zn、Ni、Se、Pb、Ca、Mg、Sr、P等14种微量元素浓度分别为10 μg/L和20 μg/L的系列标准溶液，14种元素的线性回归方程及其相关系数如表 2所示。

**2:** 14种待测元素的线性回归方程及其相关系数 The linear regression equations and correlation coefficients of 14 elements

Elements	Regression equations	Correlation coefficients
Cr	Y=1.307 1X-0.002 3	0.999 8
Fe	Y=2.545 1X+0.002 9	1.000 0
Mn	Y=1.001 2X+0.001 2	0.999 9
Al	Y=0.953 7X+0.008 2	0.998 2
Cd	Y=0.322 3X-0.000 6	1.000 0
Cu	Y=1.501 7X+0.003 2	0.999 4
Zn	Y=1.532 2X-0.001 1	0.999 6
Ni	Y=1.030 3X-0.002 1	0.999 1
Se	Y=0.370 3X-0.000 9	0.998 9
Pb	Y=0.130 7X+0.001 9	1.000 0
Ca	Y=1.388 3X+0.002 5	1.000 0
Mg	Y=1.422 5X-0.012 1	1.000 0
Sr	Y=1.522 3X-0.011 6	0.999 9
As	Y=0.375 9X+0.000 2	0.998 7

## 2 结果

### 2.1 基体效应及其校正

基体效应会对待测元素产生抑制作用，而内标法对基体效应具有补偿作用，由于In元素在样品中含量极低，电离能也与各待测元素较为接近，故而本实验选用50 μg/L的In元素作为内标，根据实测内标响应值与预期内标响应值的比值（即回收率）来校正非内标元素的响应值，有助于提高测定结果的准确性和精密度。本实验测得In元素加标回收率为97.2%-103.9%，可利用此结果对所测得的元素含量结果进行校正。

### 2.2 检出限及方法准确度

在优化的仪器条件下，以3倍信噪比计算方法计算检出限，Cr、Fe、Mn、Al、Cd、Cu、Zn、Ni、Se、Pb、Ca、Mg、Sr、P的检出限分别达到了0.063 μg/g、0.015 μg/g、0.018 μg/g、0.006 μg/g、0.056 μg/g、0.012 μg/g、0.030 μg/g、0.022 μg/g、0.015 μg/g、0.018 μg/g、0.039 μg/g、0.022 μg/g、0.026 μg/g、0.035 μg/g。为检验本方法的准确度，本文对GBW09101人发成分分析标准物质进行了同步测定，测定次数为5次，所建方法的测定值与标准值列于表 3中，从中可以看出所得结果与其参考值吻合较好，表明该方法具有较为满意的准确度和精密度。

**3:** 人发成分分析标准物质（GBW09101）的分析结果 Analytical results and reference values of 14 elements content in hair (GBW09101)

Elements	Measured values (*μ*g/g)	Standard value (*μ*g/g)	RSD (%)
Cr	4.69	4.77	-1.68
Fe	70.4	71.2	-1.12
Mn	2.95	2.94	0.340
Al	13.6	13.3	2.25
Cd	0.095	0.095	0
Cu	22.3	23	-3.04
Zn	190	189	0.529
Ni	3.75	3.71	1.08
Se	0.56	0.58	-3.45
Pb	7.3	7.2	1.38
Ca	1 072	1 090	-1.65
Mg	107	105	1.90
Sr	4.29	4.19	2.39
As	0.60	0.59	1.69

### 2.3 样品分析

按照所列的仪器条件，对48份样品中的14种元素进行了测定，测得的各类样品中14种元素含量的平均值列于表 4中。

**4:** 肺癌组与对照组样品中各元素的平均值（*μ*g/g） The average contents of 14 elements in the samples from the lung cancer group and normal group (*μ*g/g)

Elements	Lung cancer group (*n*=16)				Normal group (*n*=5)		
	Hair	Serum	Urine		Hair	Serum	Urine
Cr	0.66	30.17	0.04		0.73	31.02	0.02
Fe	13.82	1428	0.30		8.17	1112	0.37
Mn	0.71	4.21	0.01		1.69	4.49	0.01
Al	7.74	19.72	0.19		8.82	11.59	0.24
Cd	0.20	0.24	0.01		0.22	0.26	0.01
Cu	8.92	16.88	0.07		7.98	11.01	0.03
Zn	99.00	9.92	1.82		120.17	11.77	0.49
Ni	0.68	9.94	0.06		0.82	8.12	0.02
Se	1.21	69.12	0.01		8.95	73.57	0.02
Pb	3.49	11.20	0.05		2.99	8.19	0.02
Ca	689.02	88.29	5.33		701.36	127.94	8.17
Mg	68.08	20.05	6.20		112.38	26.92	11.34
Sr	2.30	21.98	0.02		5.21	18.99	0.02

## 3 讨论

ICP-MS分析过程中受到的主要干扰有同质异位素干扰、双电荷离子干扰、氧化物离子干扰以及由载气和水溶液基体中的其它原子构成的复合离子的干扰，本文待分析的14种元素的测定背景离子干扰较小。仅有^204^Pb受到^204^Hg的干扰，可通过使用干扰方程等手段进行校正。本文所选的Pb的质量数分别为208，可有效避免同质异位素的干扰。

常规上，体液和毛发中微量元素的测定一般采用加热法进行消解，在消解过程中样品容易受到污染，且难以消解完全，方法的重现性较差。而本文发展的微波消解方法消解时需要的样品和试剂量均较少，微波消解体系密闭，能够有效防止组分挥发，样品不易受到污染，样品成分可保持不变。与原子光谱法相比，本文采用ICP-MS方法对微量元素进行含量测定，具有背景干扰较小，快速、准确、灵敏度高、一次可分析多种元素的特点。

利用本研究所发展的方法对16例肺癌患者的毛发、尿液、血液中14种元素的含量进行测定，并从测定结果中发现了以下几条规律规律：①来自同一患者的不同样品中，Fe、Mn、Zn、Al、Mg五种元素的含量具有较好的线性关系；②肺癌患者组血清铜和铜与锌比值均高于正常对照组，这一现象在文献^[7]^中也有类似的报道，这一现象对于肺癌的早期诊断具有一定的临床意义。肺癌组与对照组血清样品中Cu与Zn的测定值及其比值的统计列于表 5中；③肺癌病人的毛发、血清以及尿液中Mg的含量明显低于正常人，正常人血清中Mg的含量为26.92 μg/g，肺癌患者血清中Mg的含量为20.05μg/g。一种合理的解释是：Mg在有磷酸基转移的反应中起重要作用，是维持核酸结构稳定性所必需的，Mg缺乏时核酸结构稳定性差，从而发生癌变。虽然目前尚未形成定论，但测试结果表明，肺癌病人可以适当补充Mg元素，以维持其正常水平^[8]^。这些规律对于肺癌的早期诊断和治疗效果评价具有一定的意义。

**5:** 肺癌组与对照组血清样品中Cu与Zn的测定结果（*μ*g/g） The comparison of Cu and Zn contents in the samples from the lung cancer group and normal group (*μ*g/g)

	Cu	Zn	Cu/Zn
Lung cancer group (*n*=16)	16.88±2.17	9.92 ±1.01	1.70
Normal group (*n*=5)	11.01±2.15	11.77±2.08	0.94
*t*	7.00	2.12	6.18
*P*	< 0.01	< 0.01	< 0.01

## References

[1] Gao QH, Huang KX, Fan HH (1997). The analysis of trace elements in lung cancer patients′ hair. Ai Zheng.

[2] Duan LF (2004). The diagnostic value of trace elements to the lung cancer. Chin J Misdiagnostics.

[3] Chen DD, Liu SS, Sun T (2009). The comparative research of trace elemental concentration contents in human urine from lung cancer patients and healthy people discriminant analysis. Chin J Pharm Anal.

[4] Xu XD (1995). Analysis of trace elements in hair of patients with pulmonary carcinoma. Guangdong Trace Elements Science.

[5] Zhu XL, Zhang SS, Jiang Y (2004). Analysis of seven trace element contents in benign lung tissue, lung cancer tissue and paracancerous tissue of patients with lung cancer. Chin J Lung Cancer.

[6] Zhang LL, Wu GP, Li JH (2009). Analysis of the contents of thirty elements in the plasma samples of women with lung cancer and control population. J Hygiene Res.

[7] Chen WY (1998). The change of Cu and Zn in the serum of lung cancer patients. Zhejiang Med.

[8] Cai KF, Sun GW (2007). Analysis of 6 kinds of trace elements in the serum of 120 lung cancer patients. Hebei Med.

